# Long-term aerobic exercise improves learning memory capacity and effects on oxidative stress levels and Keap1/Nrf2/GPX4 pathway in the hippocampus of APP/PS1 mice

**DOI:** 10.3389/fnins.2024.1505650

**Published:** 2024-11-26

**Authors:** Shiyan Wang, Ye Zhou, Yucheng Wu, Yali Lang, Yajun Mao, Guoyuan Pan, Zhenzhen Gao

**Affiliations:** ^1^Department of Rehabilitation Medicine, The First Affiliated Hospital of Zhejiang Chinese Medical University (Zhejiang Provincial Hospital of Chinese Medicine), Hangzhou, China; ^2^Tongde Hospital of Zhejiang Province, Hangzhou, China

**Keywords:** Alzheimer’s disease, aerobic exercise, oxidative stress, Nrf2, hippocampus

## Abstract

**Objective:**

To examine the effects of long-term aerobic exercise on oxidative stress and learning memory ability of APP/PS1 mice, focusing on the hippocampal Keap1, Nrf2, HO-1, and GPX4 proteins.

**Methods:**

Thirty APP/PS1 double transgenic AD mice were randomly divided into three groups: model group, short-term exercise group, and long-term exercise group, with 10 mice in each group. Male non-transgenic mice of the same age served as the control group. The groups underwent swimming training for 6 weeks and 12 weeks, respectively. After the intervention, cognitive abilities were assessed using the Morris water maze test. Hippocampal tissue samples were analyzed for changes in superoxide dismutase (SOD) activity and malondialdehyde (MDA) content. ROS expression was observed using dihydroethidium probe, and Keap1, Nrf2, HO-1, and GPX4 protein levels were detected by Western blot analysis.

**Results:**

Aerobic exercise significantly reduced the escape latency and increased both the time spent in the target quadrant and the number crossing the platform compared to the model group (*p* < 0.05). In the hippocampus, aerobic exercise significantly reduced the MDA content, while significantly increased SOD activity (*p* < 0.05). The level of ROS in the hippocampal region was significantly reduced by aerobic exercise (*p* < 0.05), with decreased Keap1 protein expression of and increased Nrf2, HO-1, GPX4 protein expression (*p* < 0.05).

**Conclusion:**

Aerobic exercise enhances memory and learning abilities, improves cognitive function, and reduces the oxidative stress levels in the hippocampus of AD mice, which involves in the activation of Keap1/Nrf2/GPX4 pathway.

## 1 Introduction

Alzheimer’s disease (AD), a progressive neurodegenerative disorder, is characterized by cognitive decline, memory impairment, and behavioral disturbances (Barthelemy et al., 2020). Numerous studies have identified AD as one of the most common diseases in this age group (Lane et al., 2018). In 2019, AD and other dementias were the seventh leading cause of death globally (Heron, 2021). In China, they are the fifth leading cause of death (Ren et al., 2022).

With the aging population, the prevalence of AD is increasing. Its clinical symptoms include progressive memory loss, cognitive decline, and personality changes, ultimately leading to a complete lose of independence. This progression significantly impacts the physical and mental health of patients, their families, and society. A recent study indicates that it is expected that by 2060, the number of people suffering from AD will reach 13.8 million worldwide (Association, 2022). It poses a significant burden on both patients and their caregivers. Currently, more than $818 billion is spent annually on the treatment of AD worldwide, imposing a substantial economic burden on families and society (Livingston et al., 2020).

Extensive studies on the pathogenesis of AD have implicated oxidative stress as a major factor contributing to neurodegenerative diseases and brain aging (Dhapola et al., 2024). Oxidative stress refers to an increase in the production of free radicals or reactive oxygen species (ROS) that surpasses the body’s scavenging capacity, leading to damage. It is implicated in various pathological states, including inflammation, atherosclerosis, and neurodegenerative diseases (Ionescu-Tucker and Cotman, 2021). Animal Numerous studies have shown that the pathogenesis of AD is associated with elevated levels of oxidative stress in the brain (Kheradmand et al., 2018). ROS produced by the organism play an integral role in host defense, gene transcription, and regulation of synaptic plasticity and programmed cell death. Normally, ROS can function as signaling molecules, but excessive amounts can damage to biological systems by oxidizing all major biomolecules including nucleic acids (DNA, RNA), proteins, and lipids (Mattson and Arumugam, 2018). Therefore, regulating oxidative stress levels in the brain and mitigating the ROS-induced damage may offer effective therapeutic option for AD treatment.

Due to the complexity of the mechanism leading to AD cognitive dysfunction, there is no cure for AD. Current medications only provide symptomatic relief (Yiannopoulou and Papageorgiou, 2020). Medication alone is ineffective in slowing cognitive decline in AD (Tatulian, 2022). As a result, non-pharmacological therapies, such as exercise, cognitive training, music therapy, and transcranial magnetic stimulation, are being explored. However, high-quality research on these interventions is limited. An estimated 13% of AD cases worldwide (up to 21% in the United States and 31% in Europe) may be attributed to physical inactivity (Norton et al., 2014). High levels of physical activity have been shown to delay disease progression (Santos-Lozano et al., 2016) and reduce severity (Chen et al., 2016). A study on Olympic athletes found that top-ranked swimmers exhibited the stronger thalamic-sensorimotor network connections, highlighting the impact of physical activity on brain connectivity (Huang et al., 2017). Another study indicated that aerobic exercise, compared to resistance exercise, enhances endogenous antioxidant defenses, reduces oxidative stress, and provides protection against neurodegenerative diseases (Houldsworth, 2024). While acute physical activity can increase oxidative stress, regular physical activity improves redox status (Alkadhi, 2018; Tonnies and Trushina, 2017).

The molecular mechanism through which aerobic exercise improves cognitive function in AD remains incompletely understood, necessitating further research. This study explores the effects of aerobic exercise on oxidative stress in AD, utilizing the APP/PS1 double transgenic mouse model, which is widely recognized for its ability to replicate the pathological changes of AD, making it an ideal model for such investigations (Webster et al., 2013). In this study, APP/PS1 mice were subjected to swimming training as a form of aerobic exercise. We employed the Morris water maze to assess cognitive function and used molecular experiments to analyze changes in oxidative products and antioxidant enzyme levels. By investigating the effects of different exercise durations, we aim to provide insights into how oxidative stress contributes to the pathogenesis of AD and how exercise may delay disease progression.

## 2 Materials and methods

### 2.1 Animals

Thirty male APP/PS1 double transgenic AD mice (SPF grade, 4 months old) were selected and randomly divided into three groups of 10 mice each: the model group (Model, M), the short-term exercise group (S-te), and the long-term exercise group (L-te). The results of genotype identification are presented in Supplementary Figure S1. Ten male non-transgenic mice of the same age served as the normal control group (Control, C), and purchased from ALF Biotechnology Co. (License No. SCXK2019-0004). The experimental mice were kept in the Experimental Animal Center of Zhejiang Institute of Traditional Chinese Medicine at a temperature of 25 ± 1°C and a relative humidity of 60 ± 5%. They had free access to water and were kept under a 12-h light/dark cycle. The mice in both the model group and the control group were maintained under these conditions until the end of the experiments, which lasted 12 weeks.

### 2.2 Exercise program

**Figure 1:**
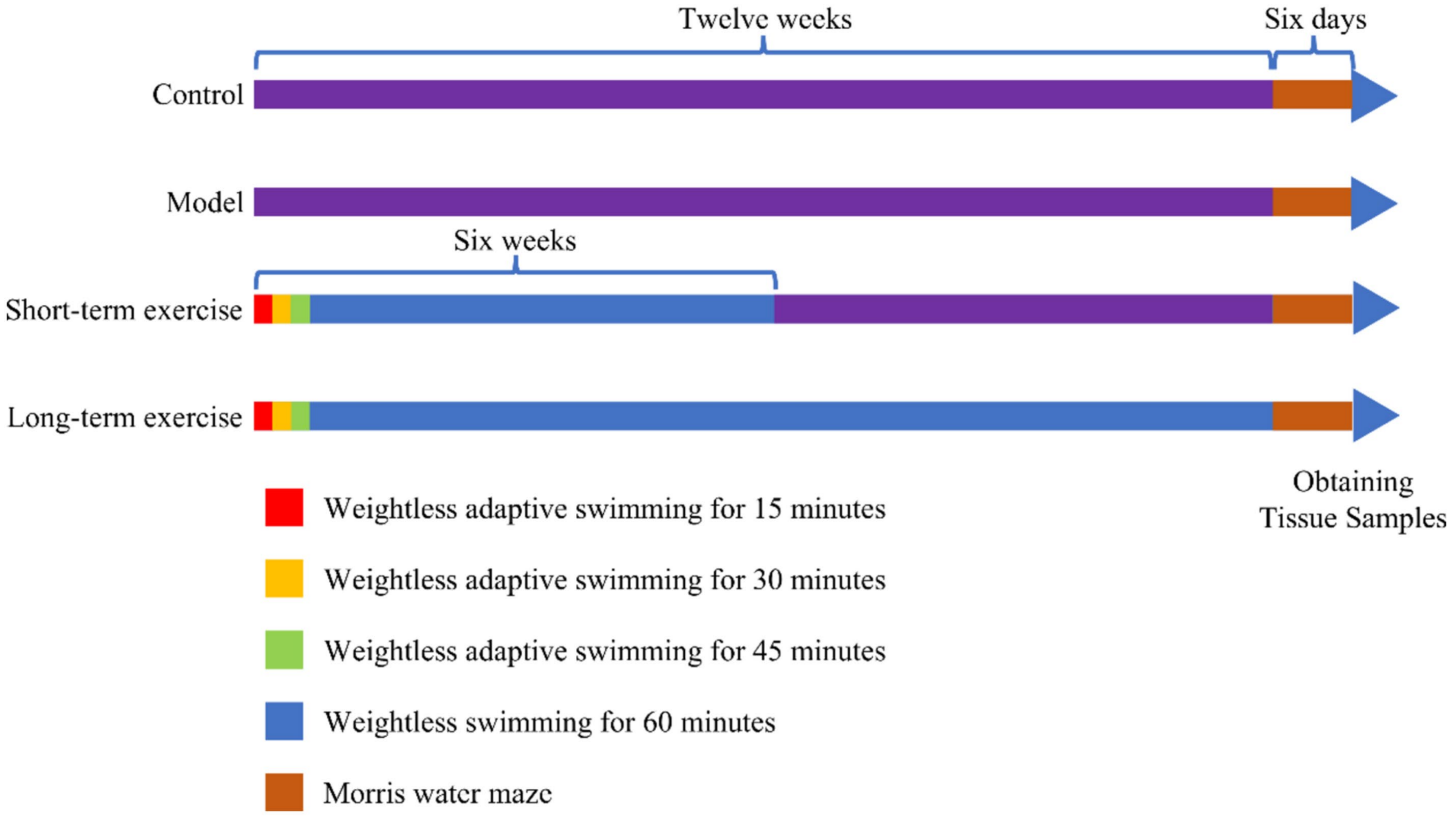
Time nodes for swimming training and behavioral testing. During the first week, the swimming exercise time was gradually increased. It began with 15 min on the first day and increased by 15 min each day, reaching 60 min by the fourth day. This 60-min duration was maintained until the end of the experiment. The short-term group (S-te group) and the long-term (L-te group) engaged in weightless swimming exercises five times a week for durations of 6 weeks and 12 weeks, respectively. A water maze test was conducted in the final week.

The M group and C group did not perform aerobic exercise (swimming). The S-te group and the L-te group swam weightlessly for 5 days a week, from Monday to Friday, with a 2-day rest period on weekends. This regimen lasts for 6 weeks in the S-te group and 12 weeks in the L-te group. During the first week, swimming time increased incrementally: starting with 15 min on the first day and increasing by 15 min each day until reaching 60 min on the fourth day. The training time was then maintained at 60 min for the remainder of the experiment (Seo et al., 2014). The water temperature was maintained at 30°C. After each training session, the rats were dried with a towel and then placed in an oven at 37°C to dry their hair before being returned to their cages. A detailed aerobic exercise regimen is shown in Figure 1.

### 2.3 Behavioral tests

The Morris water maze (MWM) experiment was initiated 24 h after the exercise intervention to assess the spatial learning and memory ability of mice. The experiment included six consecutive days of acquisition trial and 1 day of probe trial. During the acquisition trial, each mouse was gently lowered into the water from the midpoint of the four quadrants, one at a time each day, and asked to search for a circular platform submerged two centimeters underwater in quadrant IV. The maximum time search time for the platform was 120 s. If the platform was not found within 120 s, the mice were guided to the platform and remained there for 10 s. Mice that located the platform within 120 s were required to stay on it for 10 s before being retrieved. The time taken for a mouse to find the platform was recorded as the escape latency. After the acquisition trial, the underwater platform was removed, and the probe trial was conducted. For this phase, the system time was set to 120 s. Mice were placed into the water from the quadrant to quadrant IV. The number of times the mice crossed the platform and time spent in the target quadrant within 120 s was recorded.

### 2.4 Western blot

Hippocampal tissue was supplemented with pre-chilled protein lysate (50 μg/100 mg) and then collected. The tissue was fragmented by sonication, placed on ice for 30 min, and centrifuged at 15,200 *g* 4°C for 20 min. The supernatant was collected, and protein concentration was quantified using a BCA assay (Beyotime, Shanghai, China). After adjusting the concentration based on the standard curve, a loading buffer was added to denature the protein at 99°C for 8 min. The protein sample was then electrophoresed in 12% SDS-polyacrylamide gels, transferred onto the PVDF membrane, and blocked using 5% skimmed milk for 1 h. The membrane was incubated with diluted (1:1000) primary antibodies against Keap-1 antibody (ab178846; Abcam), Nrf2 antibody 28,205-1-AP; Proteintech, HO-1 antibody (13129-1-AP; Proteintech, Shanghai, China), or GPX4 antibody (14104-1-AP; Proteintech, Shanghai, China) at 4°C overnight. Beta-actin antibody (*β*-actin, 81,115-1-RR; Proteintech, Shanghai, China) serves as the internal control. Thereafter, the membranes were incubated with a secondary antibody (Goat anti-rabbit HRP, Proteintech; 1:10,000) at room temperature for 1.5 h. The membrane was washed in TBST three times for 10 min each before and after secondary antibody incubation. Immunoblot images were visualized with Image Quant LAS 4000 (GE Healthcare). The optical density values were analyzed using ImageJ. Protein quantification of Nrf2, HO-1, and GPX4 were all calculated from their respective loading control. In the figure presenting the Western blot results, for the sake of clarity and simplicity of data presentation, we selected a representative band of beta actin from among those obtained for each of the four target proteins to be shown, while the full data set including all individual beta actin bands for each protein analysis is available upon request.

### 2.5 Dihydroethidium (DHE) staining

Fresh brain tissue was fixed in 4% paraformaldehyde and subsequently stored at −80°C after trimming to avoid repeated freeze–thaw cycles. The tissue was dehydrated in a gradient of sucrose solutions and embedded in OCT compound for sectioning. A cryostat (CM1950, Leica, Germany) was used for sectioning, with the freezing chamber set to −22 to −20°C and the sample head at −18 to −21°C. Sections were washed with 1× washing buffer for 3–5 min. The DHE probe (Beyotime, Shanghai, China) was diluted 100 times with distilled water, and 100 μL of the staining solution was added to each section. The sections were incubated in a 37°C incubator in the dark for 30 min. After staining, the sections were washed with PBS three times and mounted with coverslips. The stained sections were observed under a fluorescence microscope (Leica, Germany). The presence and distribution of ROS were evaluated based on the fluorescent signal emitted by the DHE probe.

### 2.6 MDA biochemical assay

Prepare the reagents according to the instructions provided with the MDA kit (S0131S, Beyotime, Shanghai, China), add then add the samples to the reagents. Pierce a hole in the cap of centrifuge tube with a clean needle and mix the contents using a vortex mixer. Incubate the mixture in a water bath at 95°C for 40 min. Cool the tubes with running water, then centrifuge at 3,500 rpm for 10 min, and collect the supernatant. Set the spectrophotometer to 532 nm with a 1 cm optical path length, zero using distilled water, and measure absorbance value of each tube to calculate the MDA content.

### 2.7 SOD biochemical assay

Collect the supernatant of hippocampal tissue homogenate. Add the reagents and samples according to the instructions provided with the SOD kit [(Beyotime, Shanghai, China)] and mix thoroughly. Measure the absorbance at 450 nm, and calculate the SOD activity based on the absorbance readings.

### 2.8 Data analysis

All experimental data was processed using GraphPad Prism software version 8.0 (GraphPad Software, La Jolla, CA, United States). The data are expressed as mean ± standard deviation. Differences between multiple groups were analyzed using one-way ANOVA for data conforming to a normal distribution, and non-parametric tests for data that did not conform. The *p* < 0.05 was considered statistically significant.

## 3 Results

### 3.1 Effects of aerobic exercise at different times on cognitive functions of APP/PS1 mice

**Figure 2:**
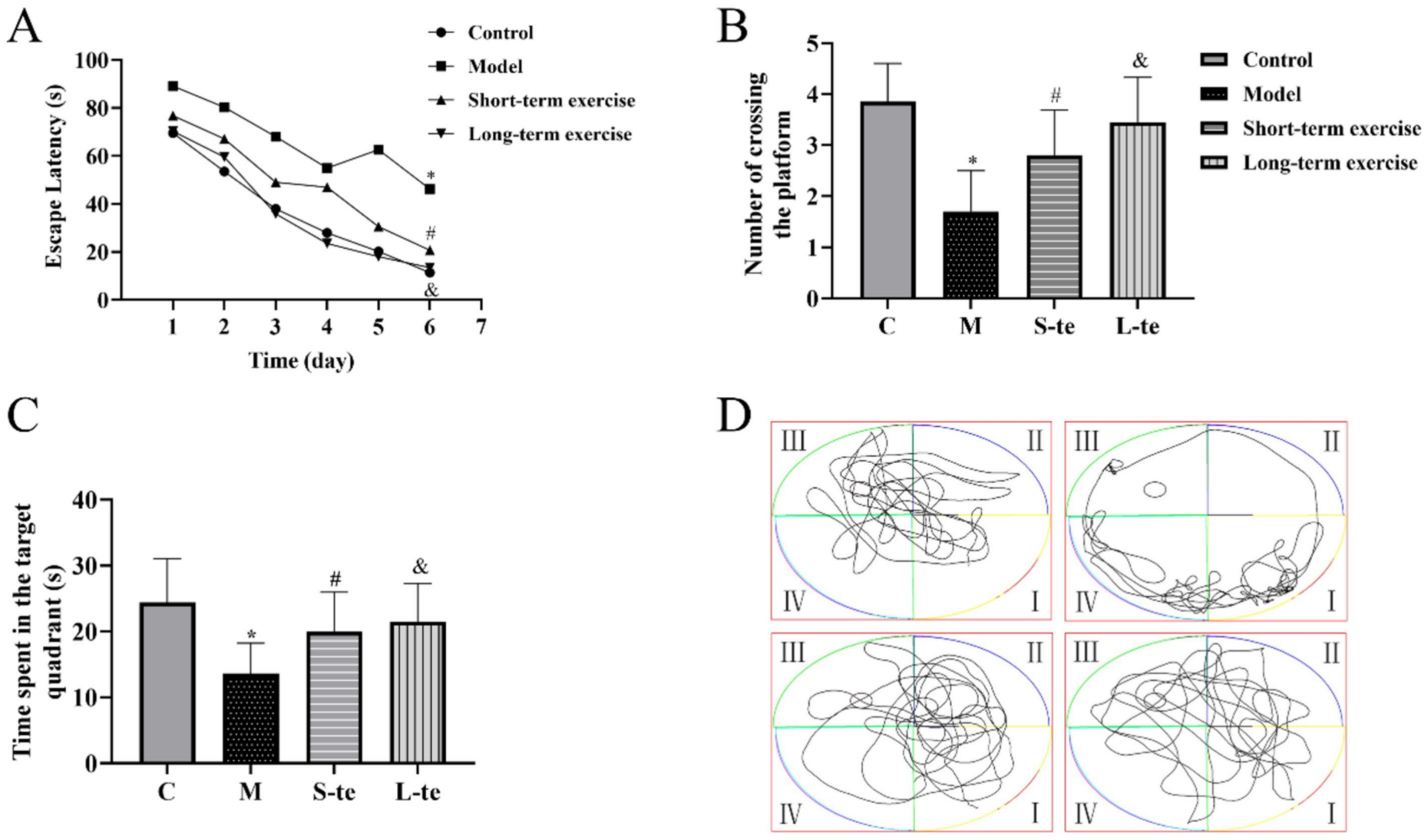
Aerobic exercise improves the spatial and long-term memory ability in APP/PS1 mice. **(A)** Average latency curve of acquisition trial per day over six consecutive days. **(B)** Time spent in the target quadrant. **(C)** Average number of times crossing the platform within 120 s. **(D)** Representative path tracking during the probe tests with a hidden platform. ∗*p* < 0.05 compared with the control group; #*p* < 0.05 compared with the model group; &*p* < 0.05 compared with the L-te group. *n* = 8, C, control group; M, model group; S-te, short-term exercise group; L-te, long-term exercise group.

In the acquisition trial, the escape latency decreased across all four groups over time. Starting on the third day, the escape latency for mice in the model group was significantly longer than that of the control group. From the fourth day, the latency in the L-te group was significantly shorter than that in the S-te group (*p* < 0.05) (Figure 2A).

In the probe trial, the AD model group showed a significant reduction in both time spent the target quadrant and the number of crossing the platform compared to the control group (*p* < 0.05). Conversely, the S-te and L-te group demonstrated a significant increase in these parameters compared to the model group (*p* < 0.05) (Figures 2B–D).

### 3.2 Effects of aerobic exercise at different times on SOD and MDA content in the hippocampus of APP/PS1 mice

**Figure 3:**
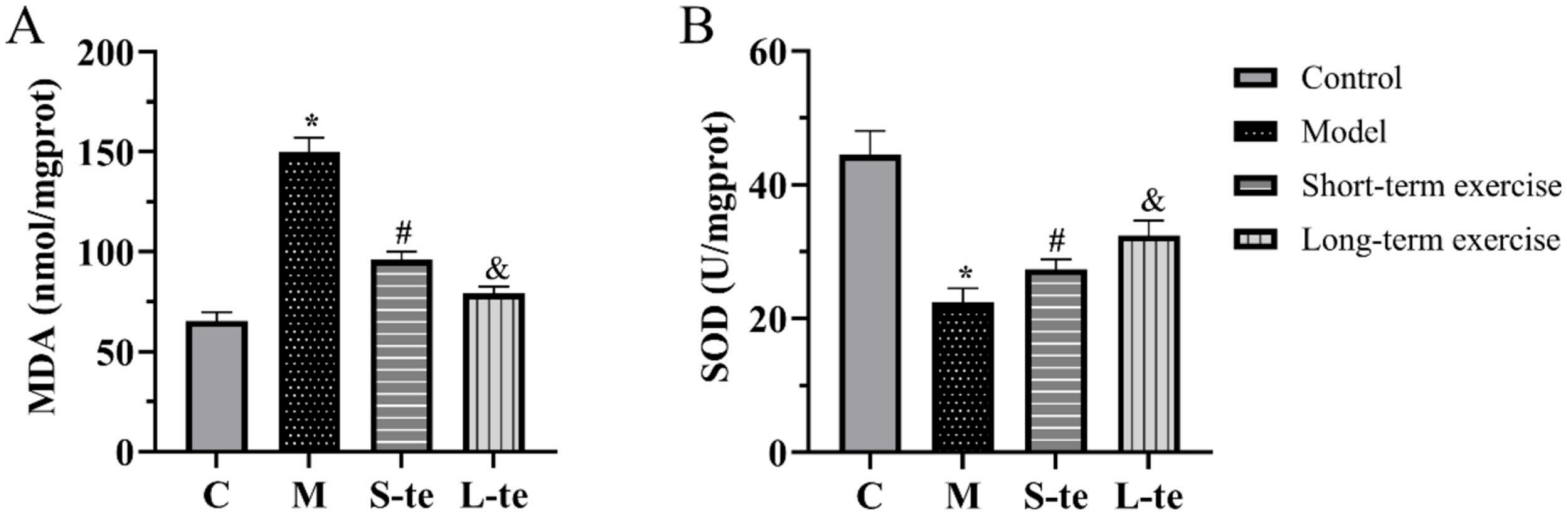
Aerobic exercise decreased MDA content and SOD activity of the hippocampus in APP/PS1 mice. **(A)** The content of malondialdehyde (MDA) **(B)** The activity of superoxide dismutase (SOD). *n* = 5, ∗*p* < 0.05, compared with the control group; #*p* < 0.05. compared with the model group; &*p* < 0.05, compared with the S-te group. C, control group; M, model group; S-te, short-term exercise group; L-te, long-term exercise group.

We detected changes in MDA content and SOD activity. The MDA assay results showed that MDA content was lower in control group. In contrast, the MDA content was significantly higher in model mice. Aerobic exercise significantly decreased the MDA content in model mice (Figure 3A). The SOD assay results showed that the SOD activity was higher in control mice. Compared to these mice, SOD activity significantly decreased in AD model group. After aerobic exercise, SOD activity was significantly higher compared to the model group (Figure 3B).

### 3.3 Effects of aerobic exercise at different times on ROS content in the hippocampus of APP/PS1 mice

**Figure 4:**
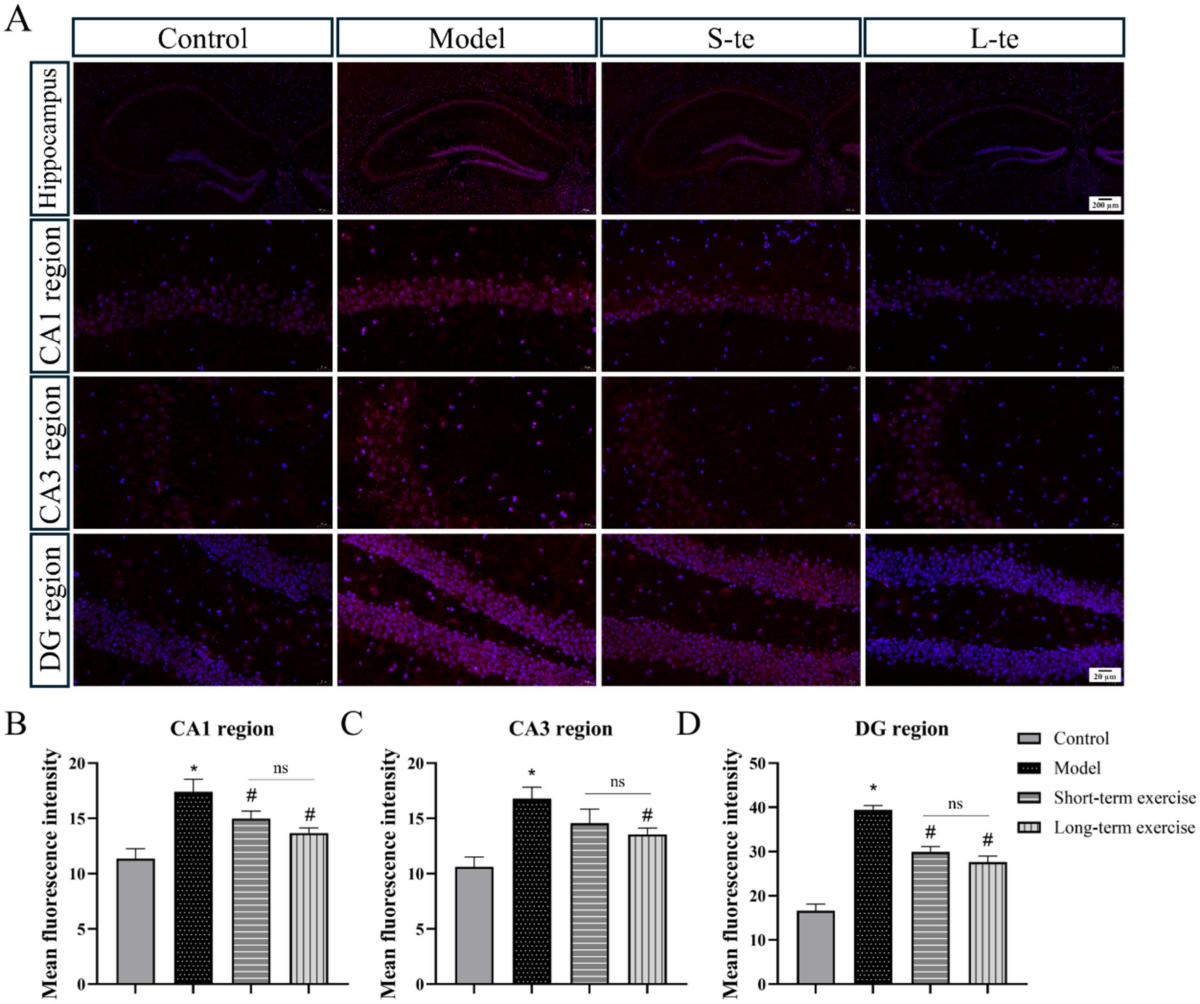
Aerobic exercise decreased ROS levels of the hippocampus region in APP/AS1 mice. **(A)** Representative immunofluorescence images of ROS in the hippocampus, including CA1 region, CA3 region, and DG region. **(B–D)** Quantitative analysis of ROS changes in hippocampal regions. *n* = 4, ∗*p* < 0.05, compared with the control group; #*p* < 0.05, compared with the model group; ns *P* > 0.05, compared with the S-te group, C, control group; M, model group; S-te, short-term exercise group; L-te, long-term exercise group.

We assessed oxidative stress by measuring ROS levels in the hippocampal region of each group. The DHE staining results indicated a significant increase in ROS levels in the CA1, CA3, and DG regions of hippocampus in the APP/PS1 model group compared to the control group (*p* < 0.05). Compared with the model group, the aerobic exercise group exhibited significantly reduced ROS levels in the hippocampus compared to the model group (*p* < 0.05) (Figure 4). Representative immunofluorescence images (×63) are presented in Supplementary Figure S2.

### 3.4 Effects of aerobic exercise at different times on Keap1/Nrf2/GPX4 pathway in the hippocampus of APP/PS1 mice

**Figure 5:**
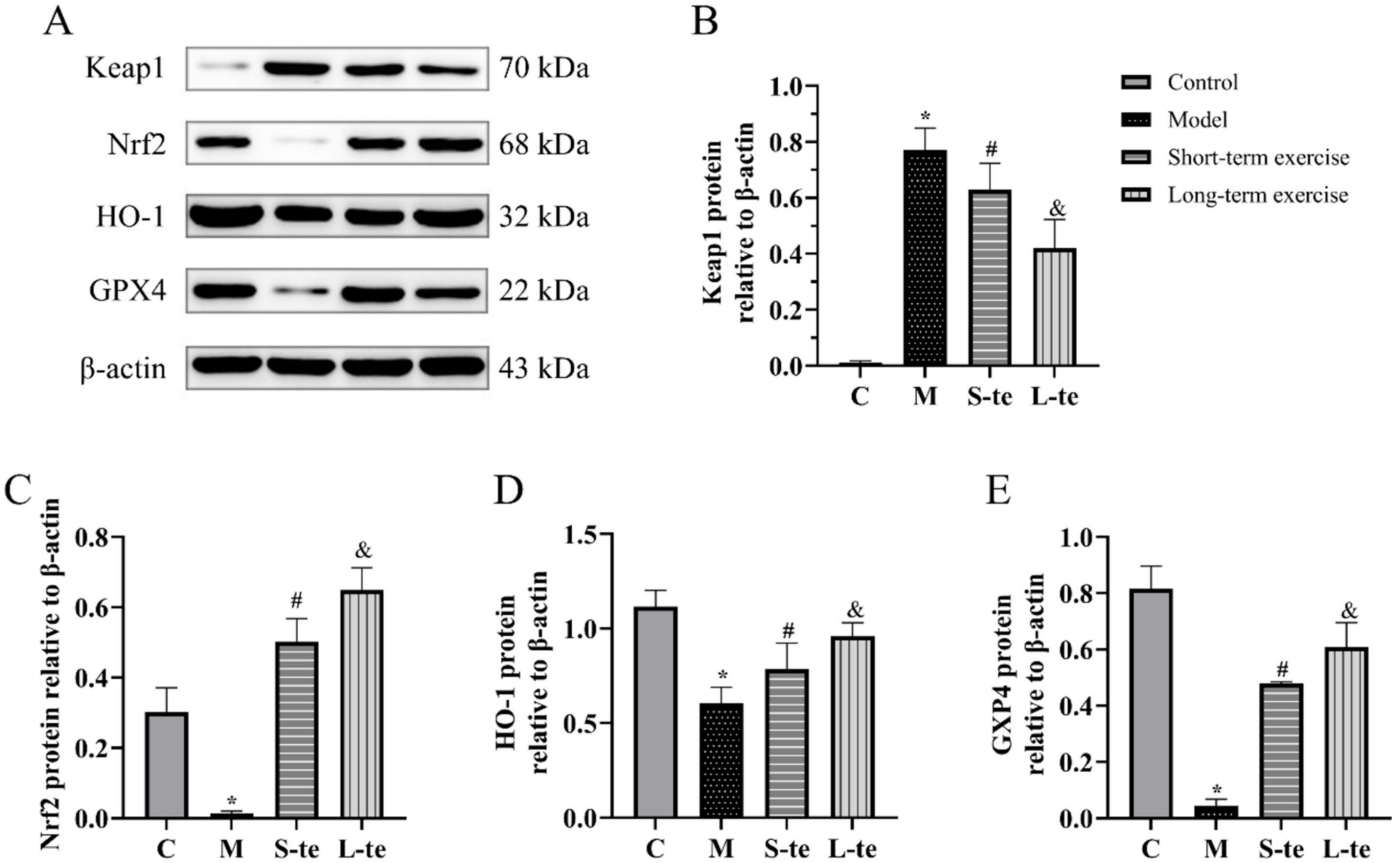
Aerobic exercise regulates Keap1/Nrf2/GPX4 pathway of the hippocampus in APP/AS1 mice. **(A)** Representative western blots of Keap1, Nrf2, HO-1, and GPX4 expression. **(B–E)** Quantification data of Keap1, Nrf2, HO-1, and GPX4 protein expression. *n* = 4, ∗*p* < 0.05, compared with the control group; #*p* < 0.05, compared with the model group; &*p* < 0.05, compared with the S-te group. C, control group; M, model group; S-te, short-term exercise group; L-te, long-term exercise group.

The protein expression of Keap1, Nrf2, HO-1, and GPX4 in the hippocampal region of brain was detected by Western blot. In the model group, the protein levels of Nrf2 and HO-1 were significantly reduced compared to the control group, while Keap1 levels, a negative regulator of Nrf2, were increased (*p* < 0.05). The aerobic exercise showed decreased Keap1 levels and increased Nrf2 and HO-1 levels compared to the model group (*p* < 0.05). Although there was no significant change, the S-te group exhibited higher expression of Keap1, and lower expression of Nrf2 and HO-1 (*p* < 0.05) (Figures 5A–D).

GPX4 is an antioxidant enzyme that is mainly responsible for reducing lipid peroxides in cell membranes, thereby protecting cells from oxidative damage induced by excessive ROS. The GPX4 expression of GPX4 was significantly reduced in model group compared to the control group. However, aerobic exercise for 6 or 12 weeks significantly increased GPX4 expression in these mice compared to the model group (*p* < 0.05). Additionally, GPX4 expression was notably higher in the L-te group compared to the S-te group (*p* < 0.05) (Figures 5A,E).

## 4 Discussion

The AD is a common neurodegenerative disease, accountings for 60–80% of dementia cases. A healthy lifestyle, particularly enhanced physical activity, is reported as a potential non-pharmacological intervention for the preventing and treating AD (Pahlavani, 2023). However, no consensus exists on the optimal exercise training methods for improving outcomes in AD patients. The findings of our study contribute significantly to the understanding of the potential benefits of long-term aerobic exercise in mitigating cognitive decline and oxidative stress in AD mouse models. By focusing on the hippocampal expression of Keap1, Nrf2, HO-1, and GPX4 proteins in APP/PS1 transgenic mice, we provide insights into the molecular mechanisms underlying the observed improvements in learning and memory capacity.

Compulsory running table exercise is generally used in animal experiments, which is difficult to realize clinically, so swimming was used as an aerobic intervention in this experiment. Our results demonstrate that both short-term and long-term aerobic exercise regimens improve cognitive function, as evidenced by reduced escape latency and increased target quadrant exploration in the Morris water maze test. However, the more pronounced effects observed in the long-term exercise group suggest a dose–response relationship, where sustained physical activity is more beneficial for cognitive enhancement. Compared to land-based exercises, aquatic exercise increases patients’ cardiac output, maximizes cerebral blood flow benefits, and improves cognitive function, including executive abilities and memory (Stanciu et al., 2023; Claassen et al., 2021). It has also been found that rats swimming daily for 5 weeks did not suffer memory impairment, even when exposed to beta-amyloid (Lourenco et al., 2019). Aquatic exercise offers a safer, low-impact environment, raising fall awareness among older adults (Moreira et al., 2020). Internationally, hydrotherapy is widely utilized for neurological rehabilitation, whereas its use is still emerging in China. By choosing swimming as an aerobic intervention, we aim to explore new clinical applications for hydrotherapy rehabilitation.

Oxidative stress, characterized by an imbalance between the production of ROS and antioxidant defenses, is a hallmark of AD pathogenesis. Under the pathological state of AD, glial cells produce large amounts of ROS. These can react with lipids and proteins on cell membrane, leading to lipid and protein peroxidation. This process forms toxic oxidative stress markers, such as protein carbonyl compounds, 4-hydroxynonenal and MDA, which increase the sensitivity of cell membrane to oxidative stress and induce neuronal apoptosis (Chen et al., 2019). Our data show that aerobic exercise significantly decreases MDA content and ROS levels in the hippocampus, indicating a reduction in oxidative damage. Concurrently, the increase in SOD activity following exercise underscores the enhancement of endogenous antioxidant enzymes, which are crucial for maintaining cellular redox homeostasis. These findings are consistent with previous studies reporting the antioxidant effects of exercise in various disease models (Henríquez-Olguin et al., 2019; Farzanegi et al., 2019).

The Keap1/Nrf2 pathway is a vital cellular defense mechanism against oxidative stress. Under normal conditions, Keap1 negatively regulates Nrf2, maintaining it in an inactive state. Upon oxidative stress, Nrf2 is released from Keap1, translocates to the nucleus, thereby upregulating the expression of antioxidant and detoxifying enzymes, including HO-1 and GPX4. Our study reveals that aerobic exercise decreases Keap1 protein expression while increasing Nrf2, HO-1, and GPX4 protein levels in the hippocampus of APP/PS1 mice. This suggests that exercise activates the Keap1/Nrf2/GPX4 pathway, promoting a robust antioxidant response that counteracts oxidative stress. The upregulation of HO-1 and GPX4 following exercise is particularly noteworthy. HO-1 catalyzes the degradation of heme into biliverdin, iron, and carbon monoxide, which have anti-inflammatory and antioxidant properties. GPX4, a glutathione peroxidase, plays a pivotal role in protecting cells from lipid peroxidation by reducing hydrogen peroxide and lipid peroxides. The increased expression of these enzymes likely contributes to the observed reduction in oxidative damage and enhancement of cognitive function. Aerobic exercise can mitigate the neurotoxicity associated with lipid oxidation (Ursini and Maiorino, 2020) and reduce oxidative stress by activating the Nrf2 antioxidant pathway in the brain (Xie et al., 2024). It modulates GPX4 levels, which converts harmful phospholipid hydroperoxides into benign phosphatidyl alcohols in a glutathione-dependent manner (Liu et al., 2023). Exercise also enhances antioxidant effects by increasing the activity of the Cu-Zn SOD enzyme. This enhancement enhances the ability of organisms to scavenge oxygen radicals *in vivo* and neutralize superoxide anion radicals *in vitro*, ultimately converting ROS into less toxic compounds (Kim, 2024). GPX4 catalyzes the reduction of glutathione to neutralize lipid peroxides and alleviate ROS-induced apoptosis or necrosis. GPX4 also is a central component of ferroptosis, which is reported to play a vital role in the pathology of AD. A recent study has shown that peroxisome proliferator-activated receptor agonists can inhibit oxidative stress and alleviate ferroptosis through the Nrf2/HO-1 signaling pathway, involving the activation of GPX4 (Qu et al., 2022). Our experimental results suggest that swimming exercise may improve cognitive function of AD mice. This improvement is potentially due to the activation of Keap1/Nrf2/GPX4 pathway, increased SOD enzyme, decreased ROS levels in the hippocampal tissue, reduced MDA production, and overall improved oxidative stress levels.

We recognize the difficulty AD patients face in maintaining consistent exercise routines due to motor function loss. To address this, tailored exercise programs that accommodate varying levels of ability, such as water-based exercises, may be more feasible for such patients. Aquatic environments can support patients with reduced mobility, offering resistance training without the strain of weight-bearing exercises. Implementing supervised exercise sessions with trained professionals can provide the necessary support and motivation for AD patients to engage in physical activity (Van der Kolk et al., 2019). This approach can help ensure that exercises are performed correctly and safely. Meanwhile, the use of assistive technologies and virtual exercise programs can offer alternative methods for engaging patients in physical activity, providing interactive and adaptable routines that can be adjusted to individual capabilities (Martinez-Martin and Cazorla, 2019; Chen et al., 2022). Besides, encouraging family members and caregivers to participate in exercise activities can provide additional motivation and support for AD patients, fostering a more consistent routine. These may encourage adherence to exercise routines and facilitate improved health outcomes.

The precise amount and duration of exercise needed remain to be clarified, which will be the next research direction we want to explore. In addition, the keap1/Nrf2/GPX4 antioxidant pathway may be related to iron ferroptosis, which needs to be proved by more molecular biological studies. While our study focused on the hippocampus, it is worth noting that the benefits of exercise may extend to other brain regions and involve additional molecular pathways. For instance, exercise has been shown to promote neurogenesis, angiogenesis, and synaptic plasticity, all of which are critical for cognitive health. Future studies investigating the broader effects of exercise on brain function and structure in AD models would be invaluable.

## 5 Conclusion

Long-term moderate aerobic exercise can improve their cognitive function via reducing oxidative stress levels in the hippocampus of APP/PS1 mice, which is associated with the activation of the Keap1/Nrf2/GPX4 pathway. This has certain clinical translational significance for delaying the progression of AD through rehabilitation training.

## Data Availability

The original contributions presented in the study are included in the article/Supplementary material, further inquiries can be directed to the corresponding authors.
