# Supplementary figures and images for: Long-term aerobic exercise improves learning memory capacity and effects on oxidative stress levels and Keap1/Nrf2/GPX4 pathway in the hippocampus of APP/PS1 mice
Source: Front Neurosci. 2024 Nov 26;18:1505650. doi: 10.3389/fnins.2024.1505650 (PMC11628528; doi:10.3389/fnins.2024.1505650)

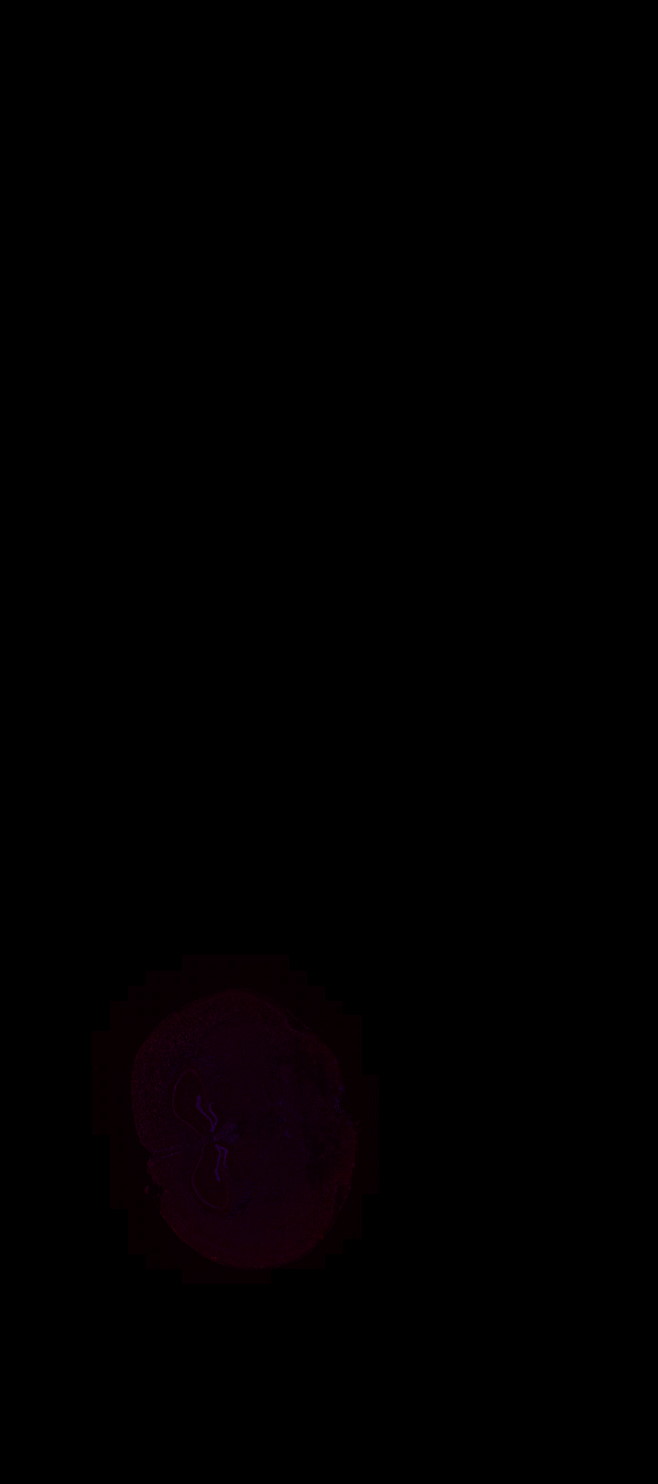

Supplement: Supplementary file 1 [file Data_Sheet_1.zip › Original Data/ROS/Control ROS(CY3).mrxs]

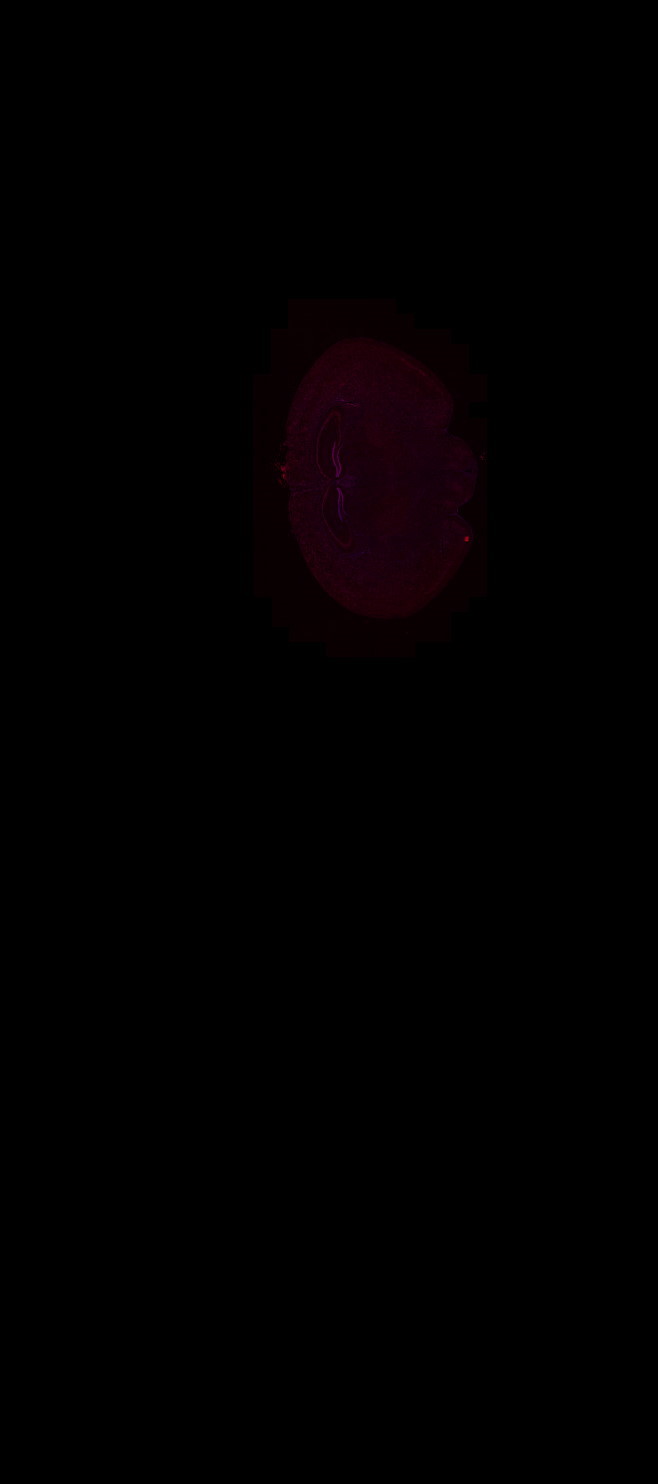

Supplement: Supplementary file 1 [file Data_Sheet_1.zip › Original Data/ROS/L-te+Model ROS(CY3).mrxs]

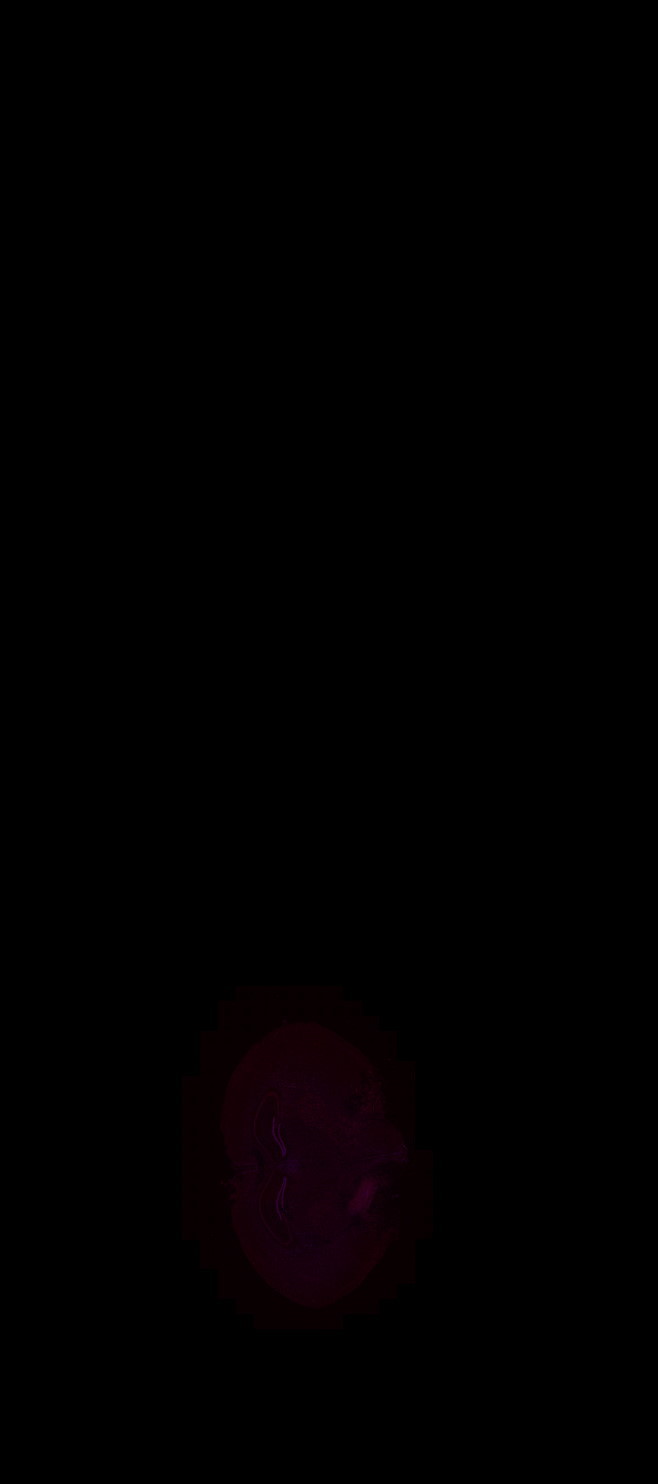

Supplement: Supplementary file 1 [file Data_Sheet_1.zip › Original Data/ROS/Model ROS(CY3).mrxs]

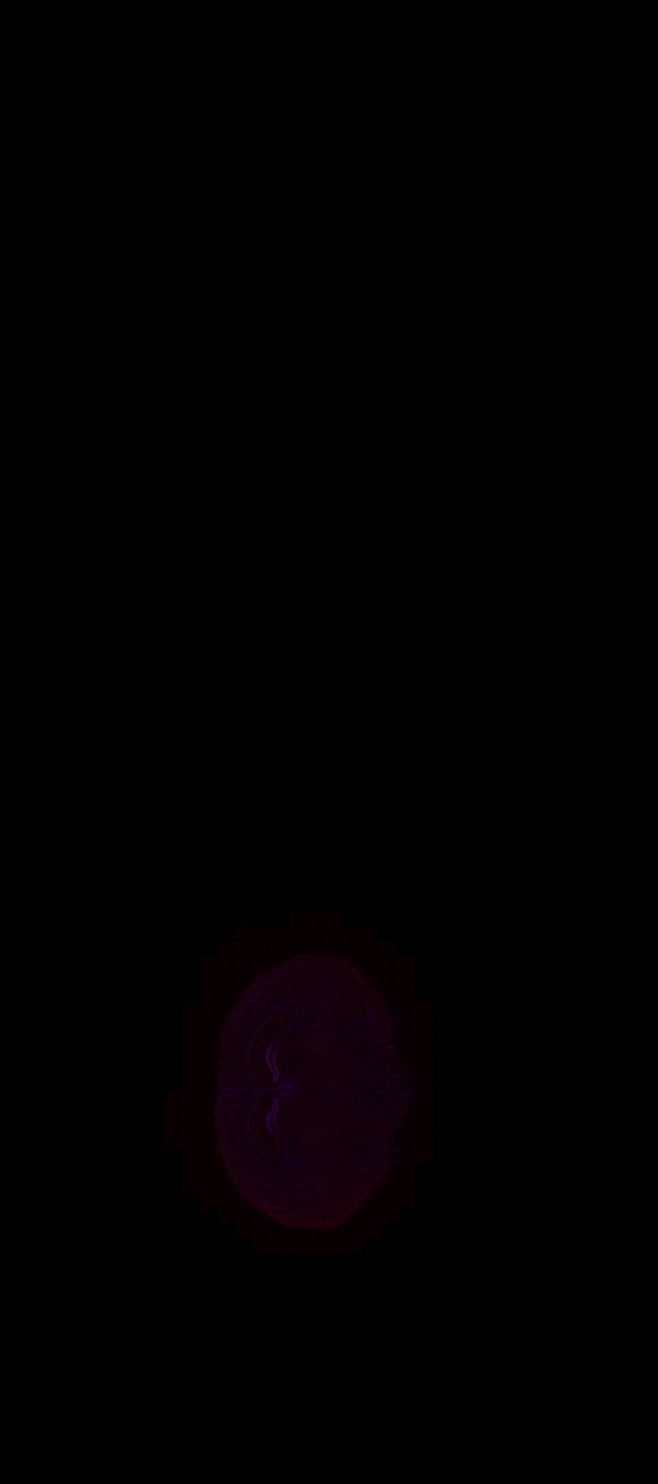

Supplement: Supplementary file 1 [file Data_Sheet_1.zip › Original Data/ROS/S-te+Model ROS(CY3).mrxs]

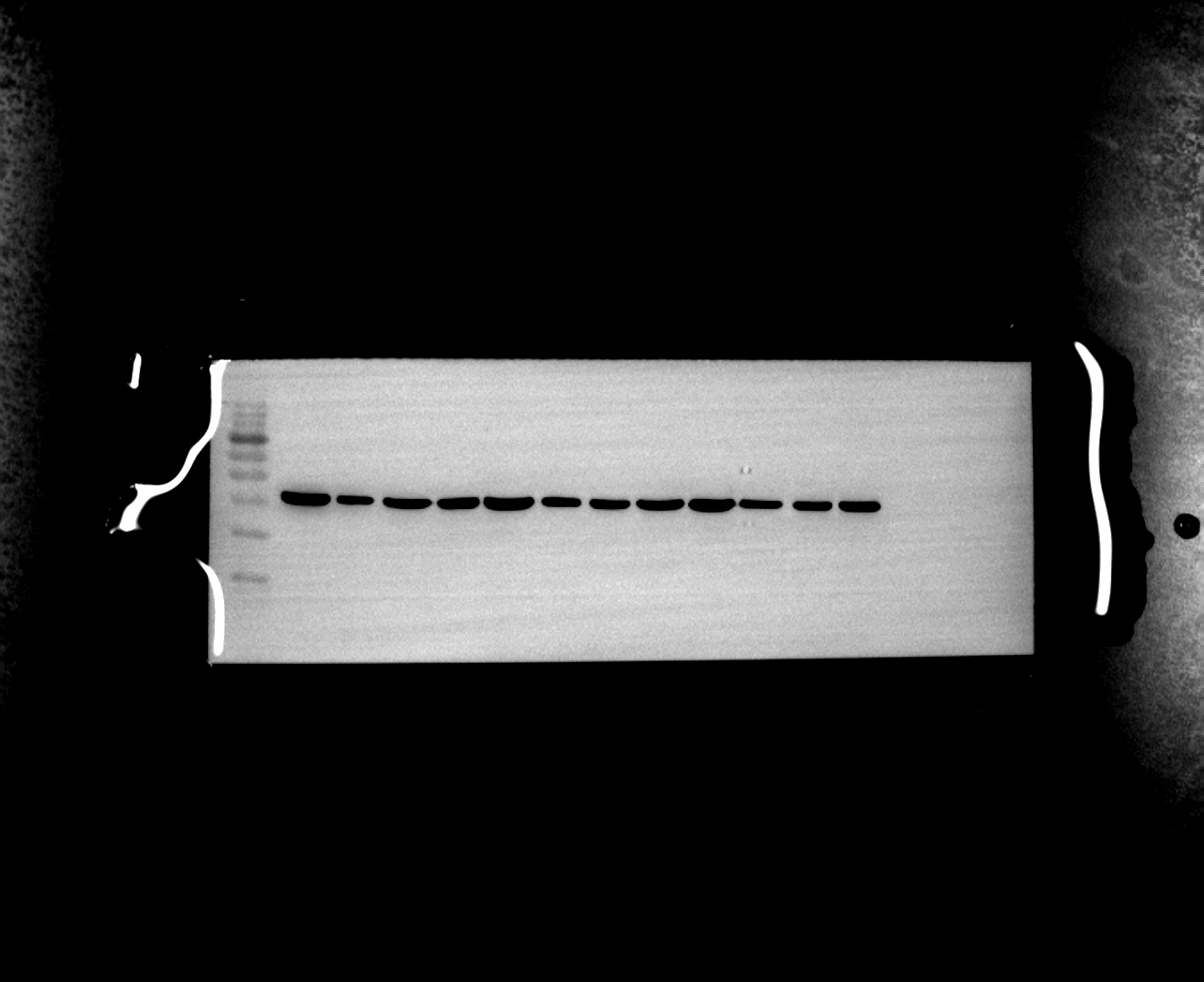

Supplement: Supplementary file 1 [file Data_Sheet_1.zip › Original Data/Western Blot/HO-1 1-2.tif]

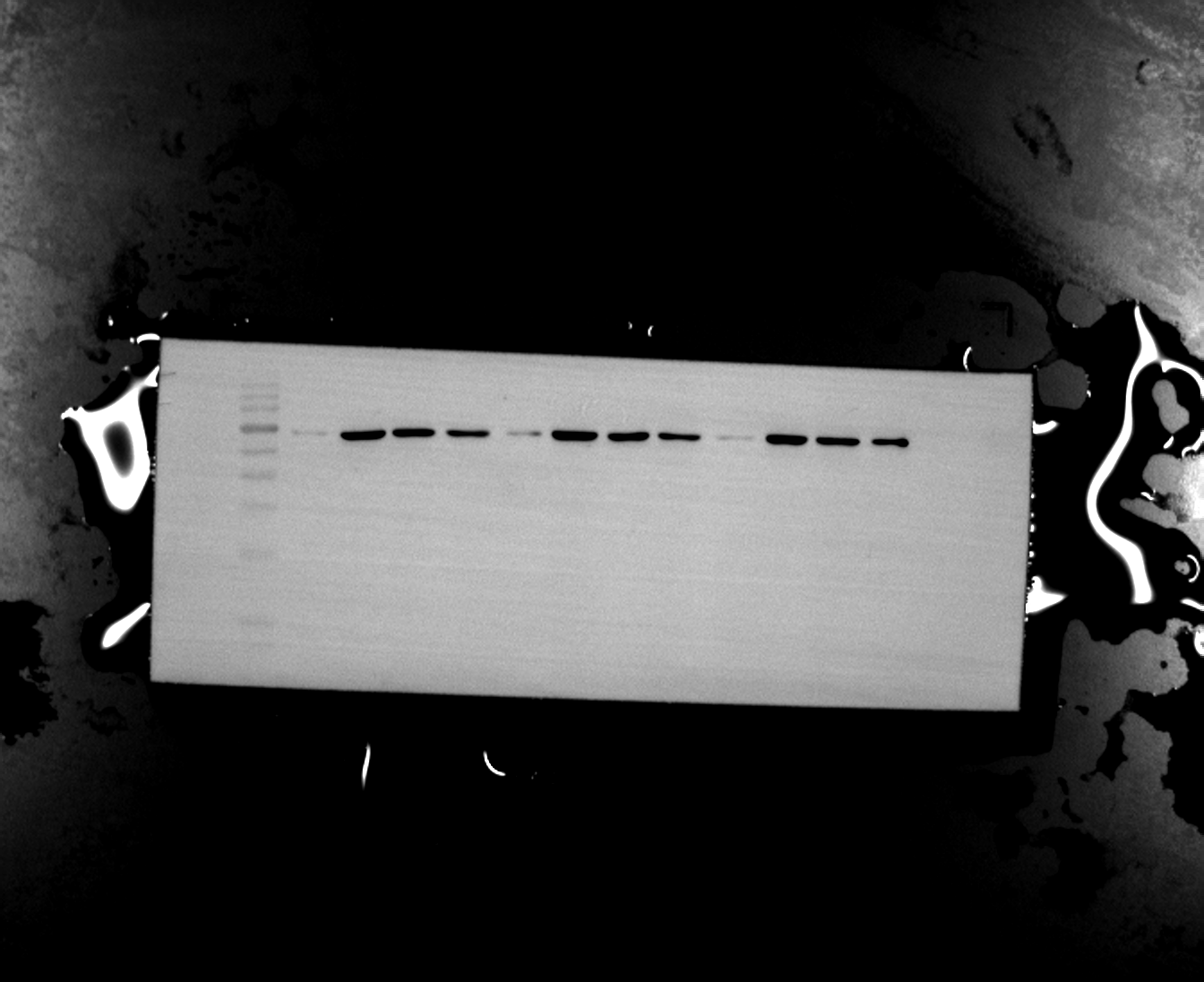

Supplement: Supplementary file 1 [file Data_Sheet_1.zip › Original Data/Western Blot/KEAP1 1-2.tif]

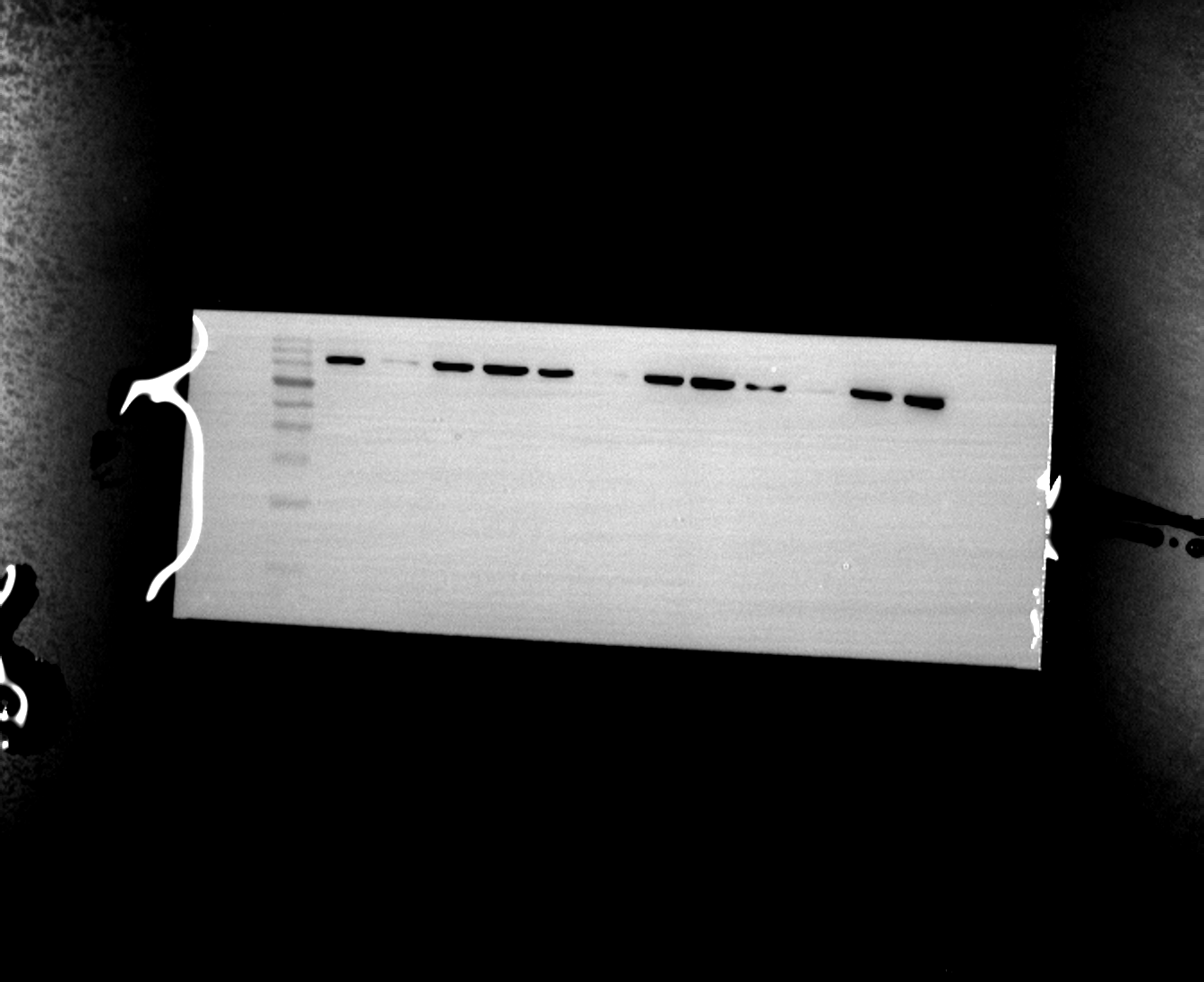

Supplement: Supplementary file 1 [file Data_Sheet_1.zip › Original Data/Western Blot/NRF2 1-2.tif]
