# Supplementary material for: Long-term aerobic exercise improves learning memory capacity and effects on oxidative stress levels and Keap1/Nrf2/GPX4 pathway in the hippocampus of APP/PS1 mice
Source: Front Neurosci. 2024 Nov 26;18:1505650. doi: 10.3389/fnins.2024.1505650 (PMC11628528; doi:10.3389/fnins.2024.1505650)
Supplement: Supplementary file 2 [file Data_Sheet_2.docx]

Long-term aerobic exercise improves learning memory capacity and effects on oxidative stress levels and Keap1/Nrf2/GPX4 pathway in the hippocampus of APP/PS1 mice

Shiyan Wang^1†^, Ye Zhou^1†^, Yucheng Wu^1^, Yali Lang^1^, Yajun Mao^1^, Guoyuan Pan^2*^, Zhenzhen Gao^1*^

^1^ the First Affiliated Hospital of Zhejiang Chinese Medical University (Zhejiang Provincial Hospital of Chinese Medicine)

^2^ Tongde Hospital of Zhejiang of Province

*Correspondence: Zhenzhen Gao ([gaofjpssw@163.com](mailto:gaofjpssw@163.com));

Guoyuan Pan (pan314500@163.com)

† These authors have contributed equally to this work

Supplementary materials

**Method**

For genotyping APP/PS1 mice, genomic DNA was extracted using the genomic extraction kit from Nantong Meijie Biological Technology Co., Ltd. (Cat No. K9053). Approximately 3mm of mouse toe was placed in a centrifuge tube with 100μl Buffer L and 10 μL Proteinase K, mixed by vortexing briefly, and centrifuged to ensure complete submersion of the tissue. The mixture was digested at 58°C until the lysate was clear. Following this, 500 μL Buffer N was added, mixed, and transferred to a DNA adsorption column. After incubating at room temperature for 2 minutes, the column was centrifuged at 10,000rpm for 30 seconds, and the waste liquid was discarded. Then, 600 μL of Buffer W2 containing absolute ethanol was added, centrifuged under the same conditions, and the waste liquid was discarded again. The column was centrifuged at 12,000 rpm for 2 minutes, placed in a clean 1.5 mL centrifuge tube, and allowed to stand for 5-10 minutes to evaporate any residual ethanol. Next, 200μl of Buffer E preheated to 65°C was added to the center of the adsorption membrane, allowed to sit at room temperature for 1 minute, and centrifuged at 10,000 rpm for 1 minute to collect the DNA solution. For agarose gel electrophoresis, a 1% agarose gel was prepared by dissolving 1-3g of agarose in 100 mL of TAE buffer, heating until melted, adding 10 μL of nucleic acid stain, and pouring into an electrophoresis mold with a comb inserted, then allowed to solidify. An appropriate amount of DNA (50 μL) and suitable DNA markers were loaded into the gel. Electrophoresis was performed at 120V for 20-30 minutes, and results were observed and photographed using a gel imaging system. The specific information on primers are listed in the Table S1.

Table S1 Specific information on primers

| Primer ID | Primer name | Primer sequence | Product | Gene name |
| --- | --- | --- | --- | --- |
|  |  | 5'- 3' |  |  |
| P025 | oIMR1597 | GACTGACCACTCGACCAGGTTCTG | T=344bp WT=None | App |
| P026 | oIMR1598 | CTTGTAAGTTGGATTCTCATATCCG |  |  |
| P027 | oIMR1644 | AATAGAGAACGGCAGGAGCA | T=608bp WT=None | Psen1 |
| P028 | oIMR1645 | GCCATGAGGGCACTAATCAT |  |  |
| P344 | mus-Internal Control-F1 | GGAGGTGAAGCAGGCTCAATC | 391bp | GAPDH |
| P345 | mus-Internal Control-R1 | GAACCAAAGCATCGACCAGT |  |  |

**Result**

The band position corresponding to the specific amplification product of the APP gene is approximately 344 bp (Fig.S1A), and that of the PS1 gene is approximately 608 bp (Fig.S1B). The simultaneous appearance of these two bands indicates that the specific fragments of the APP and PS1 genes have been successfully amplified in this mouse, and it can be determined that it has the APP/PS1 double transgenic mouse genotype. After the amplification of the APP and PS1 genes and agarose gel electrophoresis, no bands are observed on the electrophoresis pattern of wild-type mice (Fig.S1).


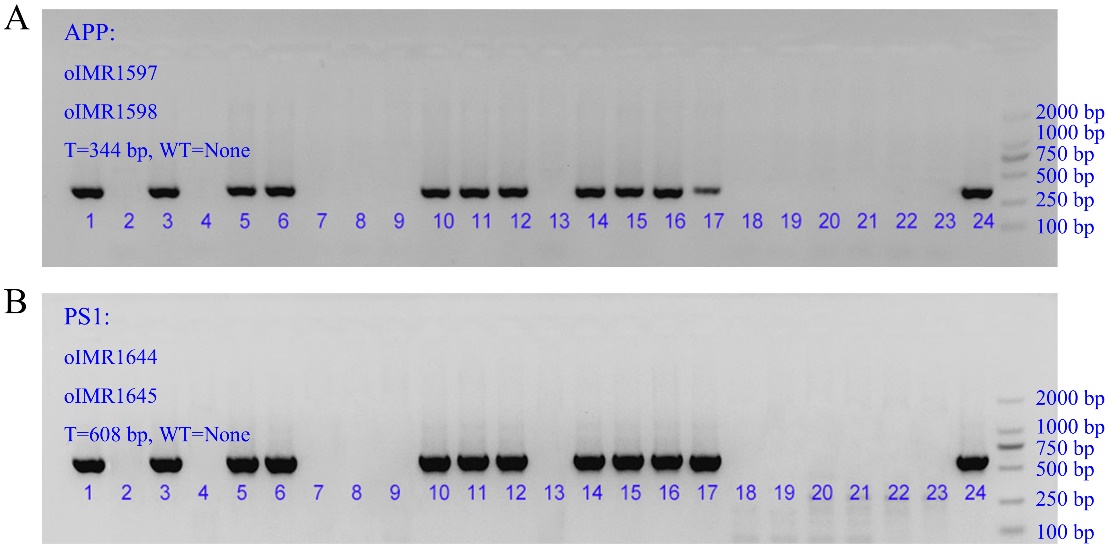


**Figure S1.** Genotype identification of APP/PS1 mice. (**A**) Representative electrophoretogram of APP. (**B**) Representative electrophoretogram of PS1.


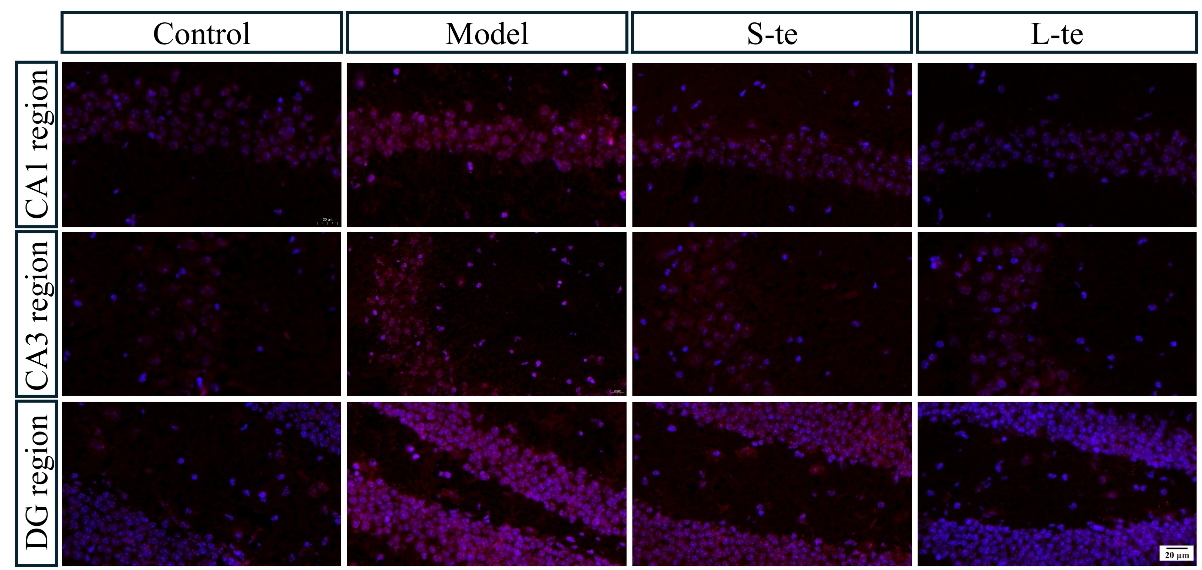


**Fig.S2** Aerobic exercise decreased ROS levels of the hippocampus region in APP/AS1 mice. Representative immunofluorescence images (×63) of ROS in the hippocampus, including CA1 region, CA3 region, and DG region.
